# Wide Dual-Band Circularly Polarized Diecletric Resonator: Innovative Integration of a Single Hybrid Feed and Thin Grounded Metasurface

**DOI:** 10.3390/mi14071432

**Published:** 2023-07-16

**Authors:** Arslan Kiyani, Mohsen Asadnia, Syed Muzahir Abbas, Karu P. Esselle, Abdelhady Mahmoud

**Affiliations:** 1 School of Engineering, Faculty of Science and Engineering, Macquarie University, Sydney, NSW 2109, Australia; arslan.kiyani@mq.edu.au (A.K.);; 2 School of Electrical and Data Engineering, University of Technology Sydney (UTS), Sydney, NSW 2007, Australia; 3 Benha Faculty of Engineering, Benha University, Benha 13512, Egypt

**Keywords:** circularly polarized, dielectric resonator antenna, metasurface, metamaterial, single-feed, compact, wideband, dual-band

## Abstract

This article presents an application of a grounded substrate-based metasurface for hosting dielectric resonators (DRs), enabling a wide dual-band circularly polarized (CP) operation. The antenna structure comprises centrally positioned rectangular DRs, one above the other, along with a 7 × 7 square-slotted metasurface. The metasurface and DRs are hosted above a grounded substrate, which is fed through a single coaxial feed placed at a specific angle, employing a modified upper probe of the coaxial feed. The proposed hybrid technique utilizes the combined benefits of the feed angle and a well-matched metasurface, resulting in performance improvement. Notably, a measured impedance bandwidth of 88.1% for $|S_{11}|$ is achieved within the frequency range of 4.0 GHz to 10.3 GHz. Furthermore, the antenna design exhibits two overlapping measured 3-dB axial ratio (AR) bandwidths: 23.62% from 4.25 GHz to 5.4 GHz and 5.12% from 7.6 GHz to 8 GHz. The peak gain of the antenna is measured at 8.4 dBic. Consequently, this innovative single-feed antenna design, characterized by its compact profile, holds significant potential for realizing multi-band operations. Furthermore, the developed antenna is well-suited for deployment in indoor radio links and INSAT applications.

## 1. Introduction

Dielectric resonator antennas (DRAs) have gained considerable attention due to their distinct advantages over microstrip patch antennas. These advantages include their light-weight construction, wide bandwidth, high gain, high radiation efficiency, reduced metallic losses, and ease of excitation [1,2]. In wireless communication systems, circularly polarized (CP) DRAs have emerged as a preferred choice compared to linearly polarized (LP) antennas [3,4]. CP DRAs offer the flexibility of utilizing either a single feed [5,6] or multiple feeds [7,8] to achieve broader bandwidths. However, the integration of power dividers and complex feeding networks in the antenna design introduces implementation complexities [9]. Traditional CP designs with single-point feeding typically exhibit a modest 3–5% 3-dB axial ratio (AR) bandwidth. To overcome this limitation, extensive research efforts have been dedicated to the exploration of diverse DR structures and enhanced feeding techniques in order to achieve wider CP bandwidths [10,11,12].

Currently, there is a noticeable research shift toward achieving CP in conjunction with multiple frequency bands. In the context of today’s advanced technology, the integration of multi-mode communications plays a crucial role in expanding system capacity to meet the growing demands of users. The increasing bandwidth requirements and higher data rates place significant strain on available spectrum resources. To efficiently use the allocated frequency bands, employing multi-band antennas offers clear advantages over their single-band counterparts. Multi-band antennas provide benefits in communication systems by assigning uplink and downlink channels to different frequency bands, thus reducing inter-channel interference. Moreover, adopting a single multi-band antenna leads to reduced overall system dimensions and cost [9,13], making CP DRAs with dual-band operation an area of considerable research interest [3,14,15,16,17,18,19,20,21]. To achieve wide dual-band CP operation, simultaneous excitation of the slot mode and DRA has been employed [19,20,21]. In the last two designs, the slot performs the dual function of exciting the DRA and radiating on its own. This technique enables the realization of a wider overlapping AR bandwidth, measuring 9.7% and 20% [19]. However, the manufacturing complexity associated with double-layered DRAs presents limitations to its widespread success. Recently, a CP DRA with a differentially inserted-fed dual-band configuration has been reported, demonstrating 3-dB AR bandwidths of 8.06% and 6.38% [22].

In recent years, there has been a surge in the popularity of metasurface-based antennas for the design of compact antenna systems [23]. Extensive efforts have been dedicated to integrating metasurface structures into antennas to enhance their performance [24,25]. By strategically placing the metasurface either above or beneath the patch/radiators, or even on a single-layer configuration, these structures exhibit unique capabilities in manipulating electromagnetic waves, thereby enabling high-gain operation and wideband characteristics [26,27]. Moreover, metasurface structures have been successfully employed to miniaturize CP microstrip single-feed antennas while improving their bandwidth [28,29]. Recently, an intriguing and innovative metasurface-based design has been demonstrated to achieve a wideband CP DRA [30,31]. However, to the best of the author’s knowledge, this technique has not yet been explored for achieving a wide dual-band CP DRA performance.

This article introduces a novel and compact metasurface-based design aimed at achieving a wide dual-band CP DRA. The proposed antenna structure incorporates a hybrid feeding technique, utilizing a simple coaxial feed with a modified upper probe placed at a specific angle. Initially, stacked DRs are employed to achieve a conventional dual-band response characterized by a relatively narrow bandwidth. To enhance the antenna’s performance, a series of square-slotted unit cell-based metasurfaces are introduced. These metasurfaces exploit the resonance properties of surface waves to generate additional resonances, thereby expanding the operational bandwidth. Through an optimization process, a 7 × 7 square-slotted metasurface configuration is obtained, resulting in a significantly broadened frequency response and improved 3-dB AR bandwidths.

## 2. Design and Analysis of a Dual-Wideband Circularly Polarized Dielectric Resonator Antenna Integrated with a Grounded Metasurface

**Figure 1:**
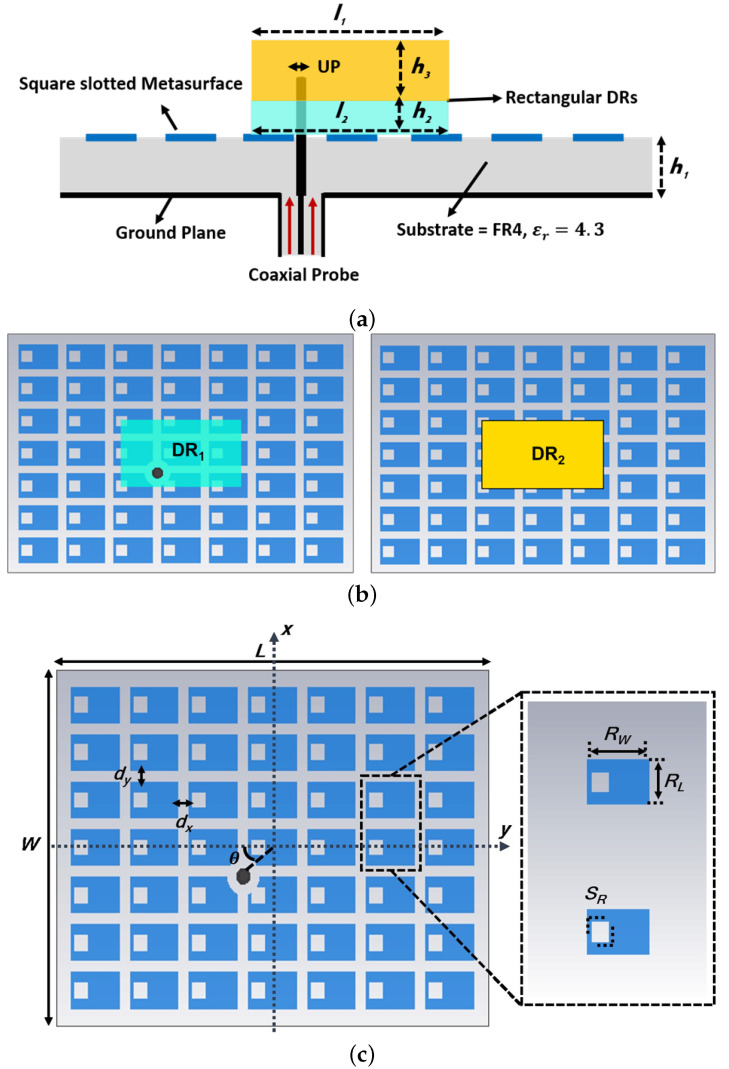
Configuration of the proposed dual-wideband CP DRA with integrated metasurface (**a**) cross-sectional view; (**b**) stacked DRs; (**c**) 7 × 7 array of square-slotted shape unit cells-based grounded metasurface.

The proposed wide dual-band CP DRA is illustrated in Figure 1a, showcasing its cross-sectional view. The antenna structure comprises a single substrate layer with a bottom-side ground plane, a top-side square-slotted metasurface, and two stacked rectangular DRs positioned above the metasurface layer. Initially, the antenna design involves solely the inclusion of the two rectangular DRs without any metasurface, as demonstrated in Figure 1b. Subsequently, periodic patches of a 7 × 7 unit cell array metasurface are introduced. The central stacking of the two rectangular DRs over the 7 × 7 unit cell-based metasurface enables dual-band operation. The dimensions of the first DR ($DR_{1}$) are defined as length $l_{2}$ = 12.35 mm and width $w_{2}$ = 22.23 mm, employing a Rogers TMM3 dielectric substrate ($h_{2}$ = 1.524 mm, $\epsilon_{r2}$ = 3.42, tanδ = 0.0027). The second DR ($DR_{2}$), with length $l_{1}$ = 12.35 mm and width $w_{1}$ = 22.23 mm, is fabricated using a Rogers TMM10i dielectric substrate ($h_{3}$ = 6.09 mm, $\epsilon_{r3}$ = 10.2, tanδ = 0.002), and it is positioned atop the first DR. The metasurface, comprising a 7 × 7 unit cell array, is implemented with a ground plane size of *L*×*W* (where *L* = 45 mm and *W* = 65 mm) on an FR4 dielectric substrate ($h_{1}$ = 3.2 mm, $\epsilon_{r1}$ = 4.3, and tanδ = 0.022), as depicted in Figure 1c. Each unit cell within the square-slotted metasurface has dimensions $R_{w}$ = 7.20 mm, $R_{L}$ = 4.65 mm, and a slot aspect ratio $S_{R}$ = 2.76 mm. The periodicity is maintained by separating each unit cell by distances dx (in the x-direction) and dy (in the y-direction). The choice of a square-slotted metasurface is motivated by its ability to provide a wideband response, distinguishing it from standard square, circular, and rectangular-shaped metasurfaces that typically exhibit narrower bandwidths.

**Figure 2:**
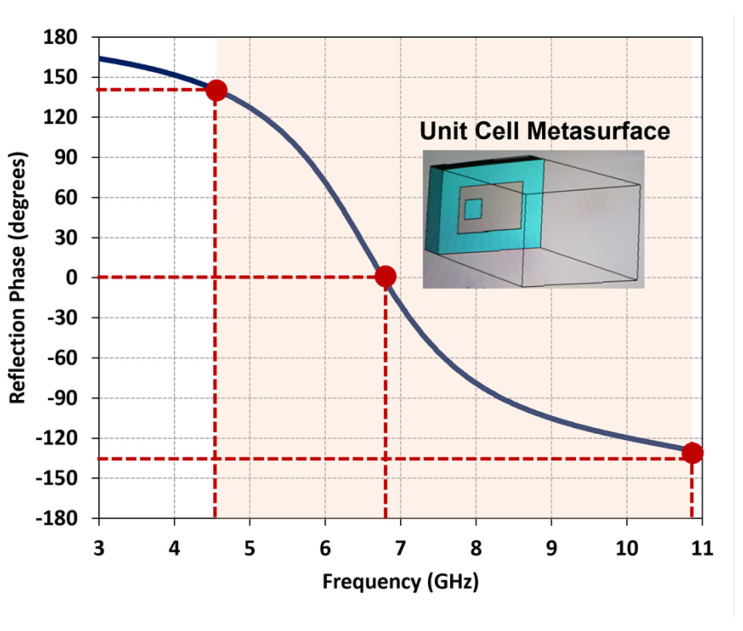
Reflection phase characteristics of unit cell metasurface.

To validate the proposed metasurface design, the reflection phase characteristics are investigated and presented in Figure 2. It is observed that the reflection phase at zero degrees occurs at 6.8 GHz. Moreover, the reflection phase bandwidth, within the range of ±135°, spans from 4.6 GHz to 10.9 GHz, demonstrating the broadband response achieved by the proposed metasurface. To ensure optimal radiation performance, the spacing between the metasurface unit cells is set to 1.55 mm in the x-direction and 1.3 mm in the y-direction. The DRs positioned above the metasurface are excited by a single coaxial feed placed at [$x_{o}$ × cos(θ), $x_{o}$ × sin(θ)], where [$x_{o}$ = 7.43 mm, θ = 29${}^{\circ }$] is the distance and angle from the center of the DR. To prevent contact between the coaxial feed and the unit cells, a metallic part surrounding the feed location is removed, creating a clearance with a radius of 2.0 mm. Through this configuration, which combines stacked DRs, a 7 × 7 metasurface, and a modified coaxial feed positioned along the diagonal line direction, a wide dual-band CP performance is optimized.

## 3. Performance Analysis and Discussion

### 3.1. Input Matching and Axial Ratio

**Figure 3:**
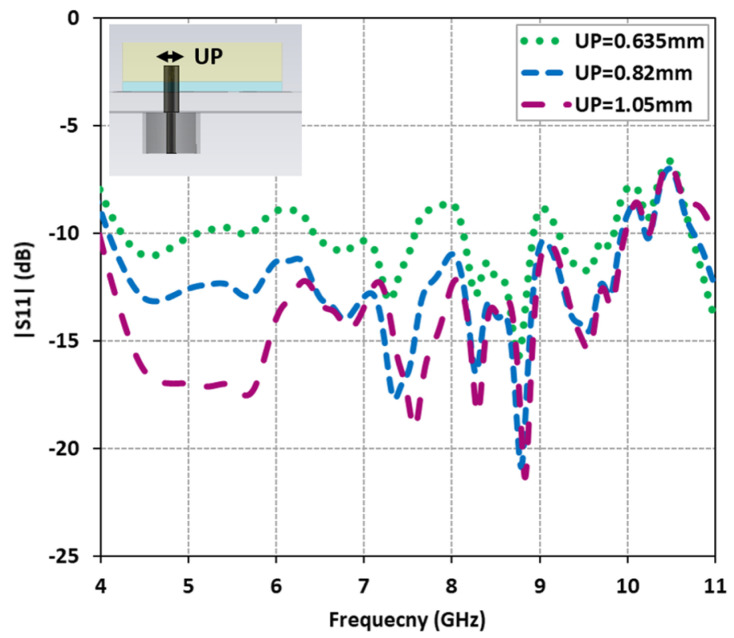
Effect of coaxial feed upper probe diameter on reflection characteristics $|S_{11}|$.

The proposed antenna design underwent a comprehensive investigation and optimization using the CST Microwave Studio. The antenna architecture adheres to the operating principle of metasurfaces, as outlined in [31]. By leveraging this principle, the design capitalizes on the surface wave resonance property of the newly devised metasurface structure to realize a compact wide dual-band CP antenna. To achieve this objective, a hybrid technique was explored, combining a diagonal probe feed for the DRA with a well-matched metasurface. This technique successfully achieved wide dual-band CP performance. The integration of two stacked DRs with strategically designed square-slotted shaped unit cells-based metasurface facilitated the generation of additional resonances in terms of both $|S_{11}|$ and AR. Fine-tuning of the upper probe of the coaxial feed allowed for control over these extra resonances. The influence of varying the upper probe diameter ($U_{P}$) was examined, and the corresponding $|S_{11}|$ results were plotted in Figure 3. The findings revealed that by tuning the upper probe diameter to 1.05 mm, a wide impedance bandwidth of 85.71% (4.0 GHz to 10.0 GHz) was achieved.

**Figure 4:**
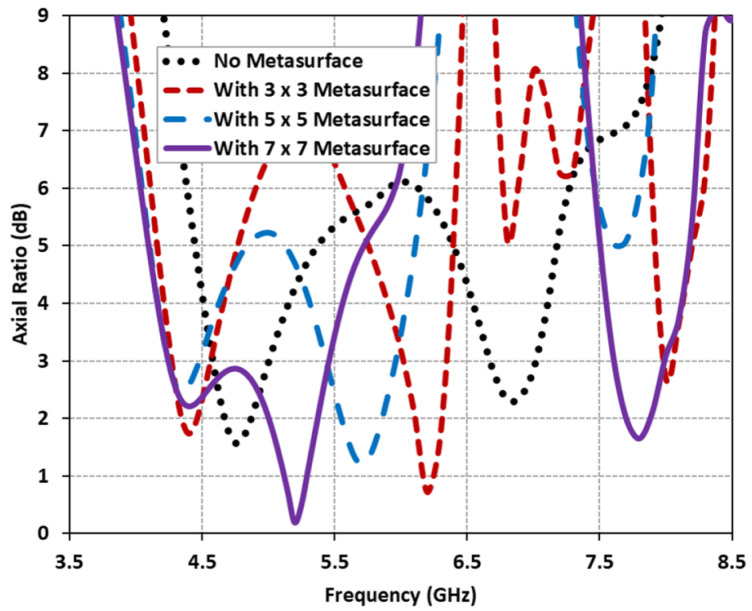
Comparative analysis of axial ratio with different unit cell metasurface configurations.

By capitalizing on the surface wave resonance property, a meticulously designed metasurface was introduced. This metasurface exhibited a multi-resonance response that was precisely controlled through the use of square-slotted unit cells. The antenna’s performance was assessed without a metasurface and subsequently with metasurfaces based on (3 × 3, 5 × 5, and 7 × 7) unit cells, while keeping all other parameters constant. The corresponding AR plots were presented in Figure 4. It was observed that the inclusion of the metasurface resulted in a multi-resonance response. In contrast, the stand-alone DRA without a metasurface exhibited two distinct resonances around 4.75 GHz (AR = 1.7 dB) and 6.8 GHz (AR = 2.3 dB). However, with the introduction of the metasurface, the antenna exhibited multiple resonances, including two resonances in the lower frequency band and one resonance in the higher frequency band. While the metasurface configurations based on (3 × 3 and 5 × 5) unit cells achieved multiple resonances, they did not meet the desired dual-band response.

**Figure 5:**
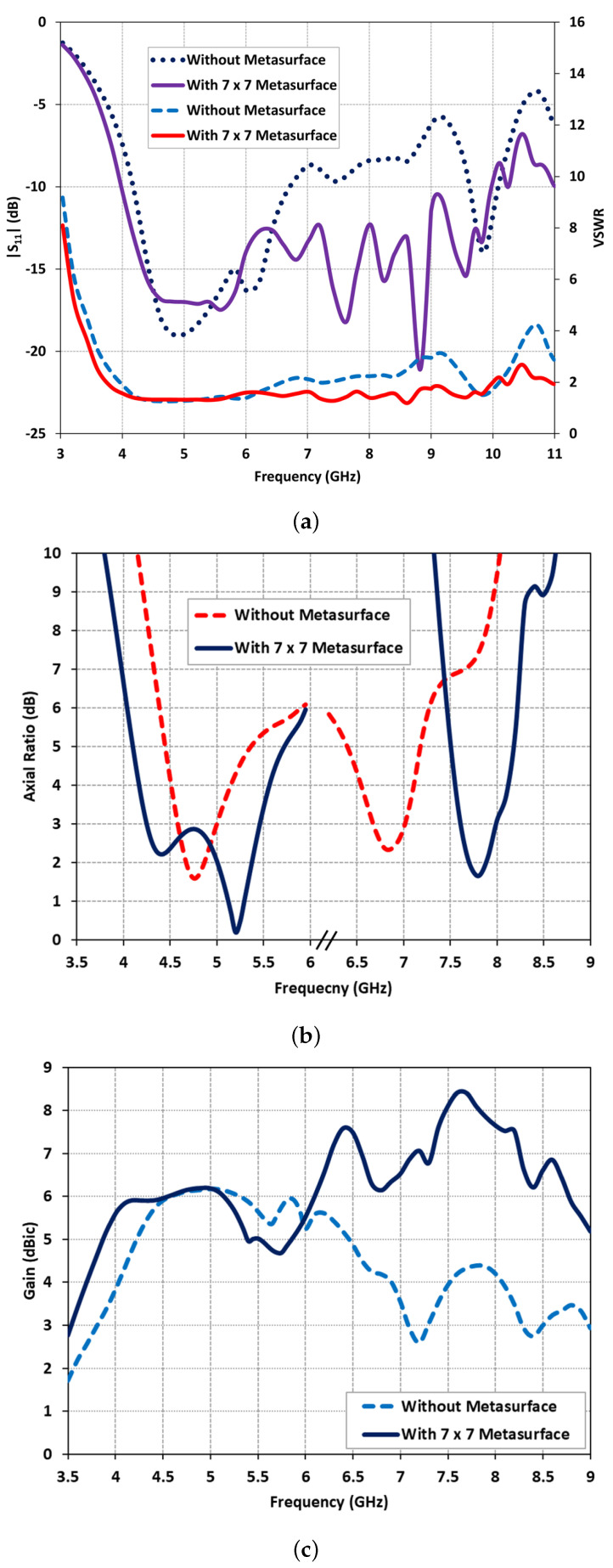
Predicted antenna performance comparison: stand-alone DRA vs. DRA with metasurface (**a**) reflection characteristics $|S_{11}|$, and VSWR; (**b**) axial ratio; (**c**) realized gain.

Hence, the optimized performance was attained by utilizing a 7 × 7 unit cell metasurface, enabling dual-band operation spanning from 4.2 GHz to 5.4 GHz and 7.62 GHz to 8 GHz, respectively. The predicted reflection characteristics and AR performance of the stand-alone DRA and the optimized DRA with the metasurface were compared and presented in Figure 5a,b. In Figure 5a, it was observed that the stand-alone DRA exhibited an impedance bandwidth of 48.14% from 4.1 GHz to 6.7 GHz and 4.87% from 9.6 GHz to 10.08 GHz. However, with the inclusion of the proposed metasurface, the impedance bandwidth expanded significantly, reaching 85.71% from 4.0 GHz to 10 GHz. This demonstrated that the DRA with the metasurface achieved a broader 2:1 voltage standing wave ratio (VSWR) bandwidth, complementing the wide impedance bandwidth. Initially, without the metasurface, the stand-alone DRA exhibited two narrow bands, characterized by single resonances around 4.75 GHz (AR = 1.59 dB) and 6.8 GHz (AR = 2.39 dB), as depicted in Figure 5b. The impact of the metasurface on the AR performance was demonstrated in Figure 5b. It was observed that the introduction of the metasurface split the first resonance into two, extending from 4.2 GHz to 5.4 GHz (with AR = 2.5 dB and AR = 0.19 dB), and shifted the second resonance to a higher frequency range from 7.62 GHz to 8 GHz (with the lowest AR = 1.65 dB).

### 3.2. Realized Gain

The realized gain of the stand-alone DRA and the DRA with a 7 × 7 square-slotted metasurface is compared in Figure 5c. The metasurface-based DRA exhibits a flat gain of approximately 6 dBic within the lower frequency band (4.2 GHz to 5.4 GHz) while maintaining a gain value above 7.5 dBic in the upper frequency band (7.3 GHz to 8.25 GHz). Figure 5c demonstrates that the introduction of the metasurface on the ground substrate results in an enhanced gain of up to 4.2 dBic within the upper frequency band.

### 3.3. Circularly Rotating Electric Field Distributions

**Figure 6:**
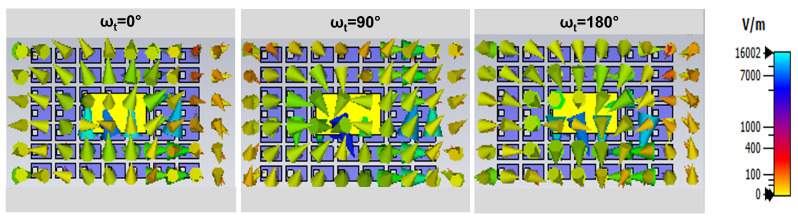
Electric field scatterings on the proposed dual-wideband CP DRA at 5.2 GHz $\omega_{t}$ = 0${}^{\circ }$; 90${}^{\circ }$; 180${}^{\circ }$.

**Figure 7:**
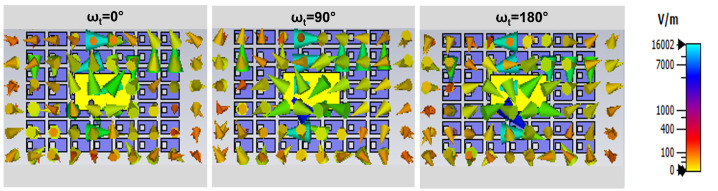
Electric field scatterings on the proposed dual-wideband CP DRA at 7.8 GHz for $\omega_{t}$ = 0${}^{\circ }$; 90${}^{\circ }$; 180${}^{\circ }$.

The electric field scatterings of the proposed metasurface-based wide dual-band DRA are analyzed to examine the generation of CP waves. The field scatterings on the antenna surface are depicted in Figure 6 and Figure 7 for two different frequencies, namely 5.2 GHz and 7.8 GHz, with $\omega_{t}$ set at 0${}^{\circ }$, 90${}^{\circ }$, and 180${}^{\circ }$. The field distributions illustrate that the circularly rotated fields exhibit a circular motion, indicating that the proposed DRA over the metasurface is capable of generating wide CP radiation. This observation confirms that the antenna satisfies the requirement for CP radiation by exciting bi-orthogonal modes. These modes exhibit nearly equal amplitudes and a 90${}^{\circ }$ phase shift across the operating bandwidth.

## 4. Fabrication, Measurement, and Validation

**Figure 8:**
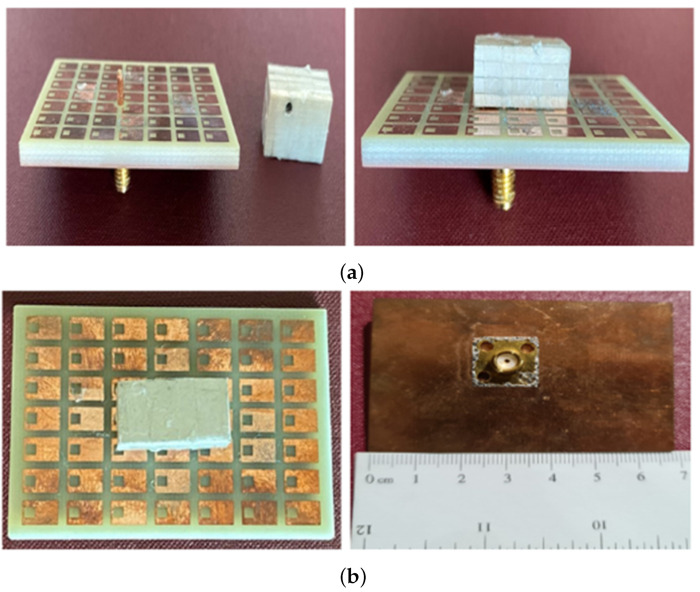
Fabricated antenna prototype (**a**) left, grounded metasurface with stacked DRs; right, front view; (**b**) left, top view; right, bottom view.

A prototype of the antenna was fabricated and is depicted in Figure 8. The antenna structure comprises a grounded substrate, two rectangular DRs, and a coaxial feed. An array of square-slotted metasurface units is etched on the top side of a grounded FR4 substrate ($\epsilon_{r}$ = 4.3, tanδ = 0.025) using the standard PCB etching technique. The first DR ($DR_{1}$) is fabricated using Rogers TMM10i dielectric substrate ($\epsilon_{r}$ = 10.2, tanδ = 0.002), while the second DR ($DR_{2}$) is made of Rogers TMM3 dielectric substrate ($\epsilon_{r}$ = 3.45, tanδ = 0.002). The heights of each substrate ($h_{1}$, $h_{2}$, and $h_{3}$) are provided in Section 2. The prototype antenna is fed using a standard 50-Ω probe feed connector. The required diameter of the upper probe feed is achieved using copper tape. The diameter of the probe feed was carefully measured using a Vernier caliper before placing the DRs over the metasurface-based substrate. Both DRs ($DR_{1}$ and $DR_{2}$) were stacked together and secured on the metasurface layer using adhesive tape, which has a negligible impact on the antenna’s radiation characteristics.

### 4.1. Measured Reflection Characteristics

**Figure 9:**
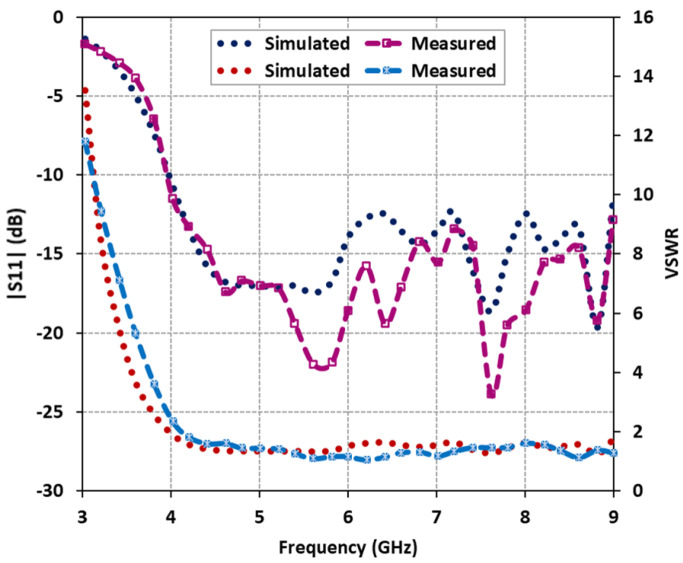
Comparison of predicted and measured antenna reflection characteristics: $|S_{11}|$ and VSWR.

The reflection characteristics, specifically the magnitude of the scattering parameter $|S_{11}|$ and voltage standing wave ratio (VSWR), were measured using an N5242A Vector Network Analyzer. The comparison between the predicted and measured reflection characteristics of the fabricated antenna is illustrated in Figure 9. The results clearly demonstrate that the proposed antenna exhibits a wide impedance and VSWR bandwidth. The predicted impedance bandwidth covers 85.71% of the frequency range from 4.0 GHz to 10 GHz for $|S_{11}|$, while the measured impedance bandwidth extends to 88.1% from 4.0 GHz to 10.3 GHz. Likewise, the predicted and measured VSWR bandwidth (with VSWR values below 2) confirms good input matching over the frequency range of 3.9 GHz to above 10 GHz. The measured reflection characteristics closely align with the predicted results, with minor discrepancies attributed to fabrication losses. These losses may arise from the presence of the bonded copper tape on the upper probe of the coaxial feed or the drilled holes in the DRs to accommodate the probe for excitation.

### 4.2. Measured Axial Ratio

**Figure 10:**
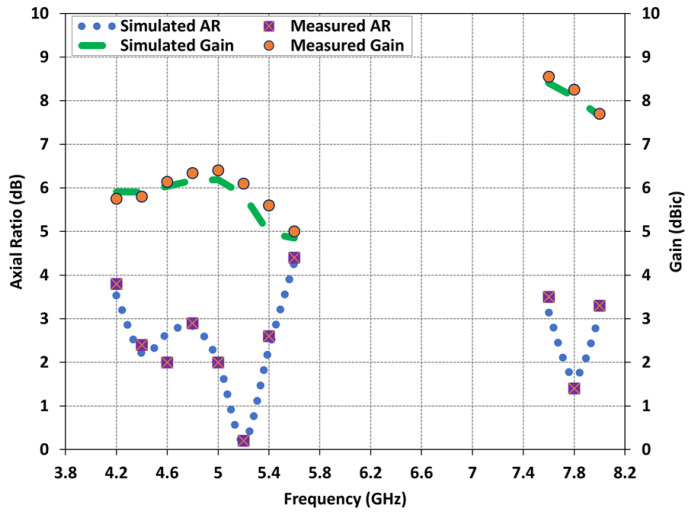
Comparison of predicted and measured antenna radiation characteristics: AR and gain.

The AR of the fabricated metasurface DRA was measured using the NSI-700S-50 spherical near-field chamber at the Australian Antenna Measurement Facility (AusAMF). The AR measurements were conducted by keeping the linearly polarized transmitting antenna fixed and measuring the received signals of the wide dual-band CP DRA while rotating it 360°. The measured AR values were collected across all frequencies to generate a plot of frequency versus AR. Figure 10 presents the comparison between the predicted and measured AR results of the antenna. The measured 3-dB AR bandwidths for the lower and upper frequency bands are determined to be 23.62% from 4.25 GHz to 5.4 GHz and 5.12% from 7.6 GHz to 8 GHz, respectively. This indicates good agreement between the predicted and measured AR plots, validating the performance of the antenna in the boresight direction.

### 4.3. Measured Gain and Radiation Patterns

**Figure 11:**
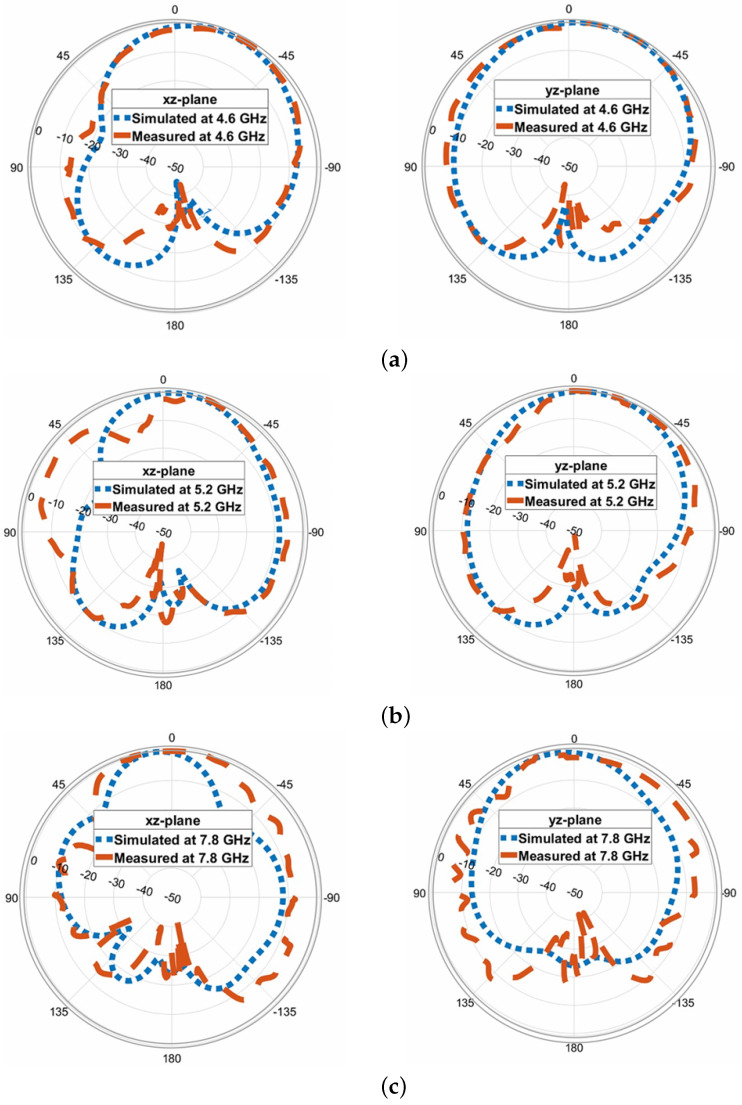
Comparison of predicted and measured antenna radiation patterns in elevation and azimuth planes at (**a**) 4.6 GHz; (**b**) 5.2 GHz; (**c**) 7.8 GHz.

In this section, we present the predicted and measured results of the proposed antenna, including the gain and radiation patterns. The peak realized gain of the antenna in the two operating bands is depicted in Figure 10. The measured gain values range from 6 dBic to 8.4 dBic across the lower and upper frequency bands, with a peak measured gain of 8.4 dBic at 7.64 GHz. At the lower frequency range of 4.3 GHz to 5.4 GHz, the measured gain values remain around 5–6 dBic, while at the higher frequency range of 7.6 GHz to 8 GHz, the gain values range from 7 to 8 dBic. A slight discrepancy can be observed between the predicted and measured results, which can be attributed to experimental errors such as antenna alignment in the anechoic chamber. The normalized predicted and measured radiation patterns of the proposed antenna are obtained in two principal planes and are illustrated in Figure 11 for three resonance frequencies: 4.6 GHz, 5.2 GHz, and 7.8 GHz. The antenna exhibits relatively stable radiation patterns in the broadside direction; however, a slight beam squinting effect is observed at higher frequencies. Overall, there is good agreement between the predicted and measured results, validating the performance of the proposed antenna.

Table 1 presents a comprehensive performance comparison between the proposed antenna and existing dual-band CP DRAs reported in the literature. It is evident from the table that the proposed antenna achieves the widest impedance bandwidth among all the compared designs. In comparison to [3,17,18], the proposed antenna excels in terms of compactness, offering a smaller profile while exhibiting a broader impedance bandwidth, wider AR bandwidth, and higher gain in both the lower and upper frequency bands. Although the designs presented in [15,16,19] have slightly lower profiles, the proposed antenna demonstrates superior radiation characteristics in terms of the compared performance parameters.

## 5. Conclusions

A novel hybrid DRA integrated with a grounded metasurface has been presented for achieving dual-wideband CP radiation. The antenna design incorporates a single coaxial feed with a modified upper probe, feeding the DRs at a specific angle. The integration of this feeding technique with a well-matched square-slotted grounded metasurface has significantly enhanced the radiation characteristics of the antenna, including impedance bandwidth, AR, and gain. To the best of the author’s knowledge, the proposed hybrid feed technique with an integrated metasurface has not been previously employed for achieving dual-wideband CP in a DRA. The fabricated prototype of the antenna has demonstrated impressive performance. The measured impedance bandwidth spans 88.1% from 4.0 GHz to 10.3 GHz, while two overlapping 3-dB AR bandwidths of 23.62% from 4.25 GHz to 5.4 GHz and 5.12% from 7.6 GHz to 8 GHz have been achieved. The antenna exhibits a peak gain of 8.4 dBic. Overall, the proposed antenna offers a compact profile and a simple configuration, which greatly reduces the complexity of the DRA feed design. Additionally, it possesses the potential for easy extension to multi-frequency band operation.

## Figures and Tables

**Table 1:** Performance comparison of the proposed DRA with existing designs in the literature.

Ref.	Impedance BW (%)	Axial Ratio BW (%)	Gain (dBi)	Antenna Profile ( L × W × h) (mm${}^{3}$)
[3]	25.3% (1.45–1.87 GHz) 35.3% (2.10–3.00 GHz)	6.3% (1.53–1.63 GHz) 3.68% (2.40–2.49 GHz)	6.09 8.09	160 × 160 × 41.1
[15]	30.07% (5.51–7.46 GHz) 7.98% (11.4–12.37 GHz)	19.98% (6.08–7.43 GHz) 3.07% (11.84–12.2 GHz)	4.9 6.4	50 × 50 × 5.08
[16]	25.7% (2.88–3.72 GHz) 9.7% (5.4–5.95 GHz)	9.52% (3.0–3.4 GHz) 5.85% (5.64–5.98 GHz)	—	50 × 50 × 13
[17]	18.9% (1.58–1.91 GHz) 7.8% (2.33–2.52 GHz)	12.4% (1.67–1.89 GHz) 7.4% (2.34–2.52 GHz)	6.23 8.01	140 × 140 × 42
[18]	23.55% (2.34–2.52 GHz) 13.33% (8.05–9.20 GHz)	14.84% (4.74–5.5 GHz) 7.11% (8.55–9.18 GHz)	4.3 5.8	100 × 100 × 10
[19]	12.2% (1.77–2.00 GHz) 21.7% (2.38–2.96 GHz)	9.7% (1.76–1.94 GHz) 20% (2.39–2.92 GHz)	—	100 × 100 × 11.6
[20]	27.7% (2.77–3.66 GHz) 8.5% (3.93–4.28 GHz)	15.7% (3.07–3.60 GHz) 6.0% (4.05–4.30 GHz)	2.3 4.7	140 × 140 × 10
[21]	11.4% (1.21–1.36 GHz) 8.4% (1.50–1.63 GHz)	14.9% (1.19–1.38 GHz) 10.1 (1.47–1.63 GHz)	5.5 4.5	100 × 100 × 55.1
[22]	30.4% (2.95–4.01 GHz) 18.5% (4.56–5.49 GHz)	8.06% (3.33–3.61 GHz) 6.38% (4.7–5.01 GHz)	6.9 7.3	60 × 60 × 9.81
Proposed DRA	88.1% (4.0–10.3 GHz)	23.62% (4.25–5.4 GHz) 5.12% (7.6–8.0 GHz)	6.15 8.4	45 × 65 × 10.81
